# The effect of paclitaxel-eluting covered metal stents versus covered metal stents in a rabbit esophageal squamous carcinoma model

**DOI:** 10.1371/journal.pone.0173262

**Published:** 2017-03-02

**Authors:** Yin Zhang, Limei Ma, Jin Huang, Jinquan Shuang, Jianping Chen, Zhining Fan

**Affiliations:** 1 Department of Digestive Disease, The First People’s Hospital of Changzhou, The Third Affiliated Hospital of Soochow University, Changzhou, Jiangsu, China; 2 Department of Digestive Endoscopy, The First Affiliated Hospital with Nanjing Medical University, Jiangsu Province Hospital, Nanjing, Jiangsu, China; Northwestern University Feinberg School of Medicine, UNITED STATES

## Abstract

**Background:**

The use of self-expanding metallic stents (SEMSs) is the current treatment of choice for malignant gastrointestinal obstructions. However, these stents can promote only drainage and have no antitumor effect. Some studies have reported that drug-eluting SEMSs may have tumor inhibition potential. The aim of this study was to evaluate the efficiency and safety of paclitaxel-eluting SEMSs (PEMSs) in rabbit esophageal cancer models.

**Materials and methods:**

A PEMS was covered with a paclitaxel-incorporated membrane, in which the concentration of paclitaxel was 10% (wt/vol). The rabbit models were created endoscopically. Then, a PEMS or SEMS was endoscopically inserted into the rabbit esophagus. Two weeks after stent placement, the rabbits were sacrificed, and we evaluated the tumor volume, area of the wall defect, area of the tumor under endoscopic ultrasound (EUS) before and after stent placement, status of the proximal esophageal obstruction, tumor metastasis food-intake and weight loss.

**Results:**

A total of 26 rabbits received stent insertion and survived until sacrifice, and migration occurred in 4 cases, 3 in SEMS group and 1 in PEMS group. For the remaining 22 rabbits, at the sacrificed time, the average tumor volume was 7.00±4.30 cm^3^ in the SEMS group and 0.94±1.51 cm^3^ in the PEMS group (*P*<0.05). The area of the esophageal wall defect was 0.70±0.63 cm^2^ in the SEMS group and 0.17±0.16 cm^2^ in the PEMS group (*P*<0.05). The tumor area under EUS was 4.40±1.47 cm^2^ in the SEMS group and 1.30±1.06 cm^2^ in the PEMS group (*P*<0.05). At the time of stent placement, tumor area under EUS was comparable in the two groups. Other indices did not significantly differ between the two groups.

**Conclusions:**

SEMS and PEMS are both safe and effective to relieve dysphagia in rabbit esophageal cancer models. A PEMS can serve as an alternative tool for advanced esophageal cancer that may inhibit tumor growth by serving as a drug sustained-release platform. Clinical trials of the stent are warranted in the future.

## Introduction

Esophageal cancer is the 8^th^ most common cancer worldwide and is increasing in incidence [1,2]. Patients with esophageal carcinoma always have a poor prognosis because of dysphagia due to malignant obstruction, which also leads to malnutrition, dyscrasia and reduced quality of life [3]. Non-operative management by metallic stent insertion is generally used because stent deployment is less invasive than surgery. Moreover, survival time is increased, and patient quality of life also improves [4]. However, conventional stents can facilitate only palliative relief and have no antitumor effect. Re-occlusion after self-expanding metallic stents (SEMSs) insertion can occur as a result of tumor ingrowth, tumor overgrowth and hyperplasia of the esophageal epithelium, especially at the two ends of the stent [5]. In recent years, drug-eluting stents have been widely used for stenosis in cardiovascular diseases, uploading anti-proliferative drugs [6,7]. We hypothesize that drug-eluting stents may inhibit tumor growth if uploading anti-tumor drugs.

As a new antineoplastic agent, paclitaxel is currently being used to treat several types of cancer. The drug inhibits tumor growth by binding to β-tubulin and stabilizing polymerized microtubules [8,9]. Paclitaxel is effective at inhibiting cell proliferation in human gallbladder carcinoma, pancreatic adenocarcinoma and esophageal carcinoma [10]. Thus, paclitaxel-eluting SEMSs (PEMSs) may have antitumor effects against or prevent tumor overgrowth of malignant esophageal strictures [11–13]. The local controlled-release platform is safe as it offers local drug delivery and prevents systemic adverse reactions, which was also confirmed by Wang [14]. The aim of this study was to evaluate the efficiency and safety of PEMSs in a rabbit esophageal cancer model by analyzing the size of the tumor, status of the proximal esophageal occlusion and adverse events.

## Materials and methods

### Preparation of PEMSs

SEMSs (Niti-S polyurethane-covered stent, Garson, China) were 20 mm long, 6 mm wide in the middle and 8 mm in wide at the proximal end when fully expanded and mounted on a 6F stent introducer set. The average diameter of the rabbit esophagus was approximately 4–5 mm in normal cases and even narrower in cases with cancer. In our previous study, the diameter of stent was wider, which was 8 mm in the middle with partially covered membrane. After the stent insertion, less food intake and proximal esophageal occlusion occurred [15]. Thus, the diameter of the stent was established as 6mm. The PEMS was uploaded with 10% (wt/vol) paclitaxel (Taxol, Hongdoushan, China) by the State Key Laboratory of Pharmaceutical Biotechnology, School of Life Sciences, Nanjing University, China. Based on specified indices, such as the drug-release rate and the effect of the drug on the mucosa, a PEMS with 10% paclitaxel was the first choice of treatment.

### Animal model

All experimental procedures were approved by the Committee on Animal Research at The First Affiliated Hospital with Nanjing Medical University and The First People’s Hospital of Changzhou. The study was carried out in strict accordance with the recommendations in the guide for the care and use of laboratory animals of the national institutes of health.

Male or female New Zealand White rabbits (Jiangsu Agricultural Academy of Science, China) weighing between 2.0–2.5 kg, were used in this study. They were allowed free access to food and water under the temperature of 25°C and regular day-night cycle. Each rabbit was anesthetized by intraperitoneal injection with pentobarbital sodium (35 mg/kg, Sigma Chemical Co., St. Louis, MO, USA) when they underwent tumor implantation and stent insertion to minimize suffering.

Tumor propagation: Tumor propagation was maintained by injection into the hind limb gluteal muscles of the rabbits. Before the study, VX2 tumor tissue were got from Prof. Yi Miao at the First affiliated Hospital with Nanjing Medical University (About 20 small pieces in 2ml frozen stock solution). The tissue was stored at -80°C and recovered for injection in our lab. The tissue was cut into small pieces (the diameter <1mm and the volume about 0.5mm^3^) and then injected into the hind limb gluteal muscles of 2 rabbits. After 2–3 weeks, the rabbits were euthanized. The VX2 tumor was harvested and also cut into small pieces (about 0.5mm^3^) in an asepsis environment.

The storage of the tumor tissue: 4 pieces of tumor fragments were preserved in 0.5ml of refrigerating fluid (10% DMSO and 90% FBS) in a 2-ml freezing tube. The tissue was placed at 4°C for 20 min, -20°C for 2 h and then stored at -80°C. The storage time was less than half a year to ensure the activity of the tumor fragments. For the next time of tumor propagation or endoscopic injection, the VX2 tumor tissue was recovered, and 4 pieces of tumor fragments were preserved in 0.5ml saline solution in a 2-ml syringe at 0°C.

Endoscopic implantation: Animals were fasted with free access to water for 24 h. After anesthesia, each animal was placed in the left lateral decubitus position. An ultra-slim endoscope (GIFXP260, Olympus, Japan) was inserted into the esophagus, endoscopic submucosal injection with about 1 ml solution (0.01% indigo carmine + 0.01% epinephrine + 0.9% saline) was performed and then the tumor fragments were injected into the submucosal layer of the thoracic esophagus. After the endoscopic implantation, the animals were fasted for a further 24 h prior to reintroduction of their usual diet. 80000 units of penicillin were intramuscular injected regularly for 3 days. The method of tumor propagation and endoscopic implantation was the same as it of our previous study [16] and it was proven to be effective.

After endoscopic implantation of VX2 tumor tissue, endoscopy (Choledochoscope, CHF-P20, Olympus, Japan) was performed every week to monitor the tumor growth and degree of esophageal stenosis. Food intake and weight were also monitored every day. Severe stricture (esophageal stenosis under endoscopy greater than 2/3 of the luminal diameter) was observed in all rabbits at 2 or 3 weeks after tumor implantation. When endoscopy revealed the severe stricture, the animal was a candidate for stent insertion. The rabbits were randomly enrolled in the PEMS group or SEMS group.

### Stent placement

**Fig 1:**
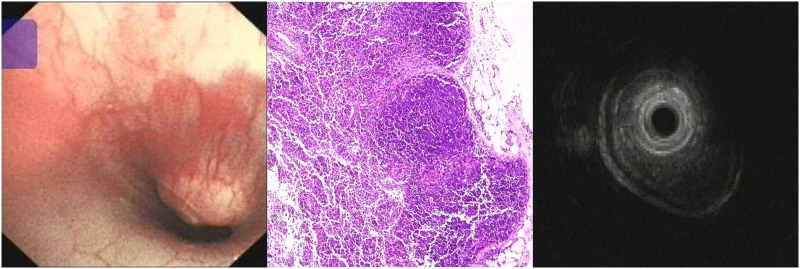
Characteristics of esophageal squamous carcinoma before stent implantation. (A) Endoscopic view of severe stricture of the lower esophagus. (B) H&E staining view: Carcinoma cells and inflammatory cells were observed throughout the specimen. (C) Endoscopic ultrasound view of the tumor with a smooth serosa.

**Fig 2:**
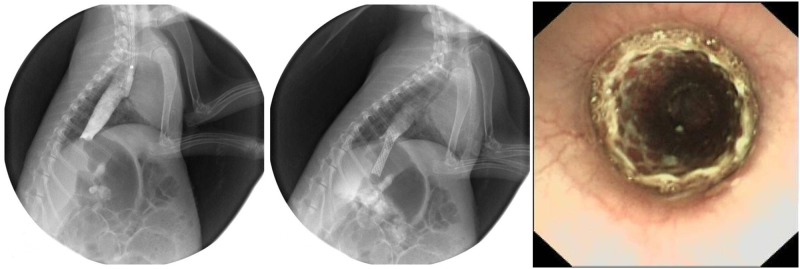
Procedure of stent implantation. (A) Contrast was injected into the esophagus before stent insertion, which revealed dilation of the upper esophagus. (B) Contrast medium was injected into the esophagus after stent deployment, thus confirming the stent covering the stricture. (C) Endoscopic view showing the stent *in situ*.

The rabbits received the similar preoperative preparation, including the fasting time and anesthesia. A choledochoscope was perorally inserted and confirmed the severe stricture of the esophagus. Then, an ultrasound endoscope (UM-DP12-25R, Olympus, Japan) was inserted to confirm the position and size of the tumor in each rabbit (S = length*width). A SEMS or PEMS was introduced into the esophagus with an introducer set. Before the introducer reached the correct site, 0.5 ml contrast medium (Iohexol solution, GE healthcare, China) was injected into the esophagus to reconfirm the exact position of the stricture. Then, the stent was deployed at the supposed site, covering the stricture (Figs 1 and 2). All endoscopic procedures were performed by two experienced endoscopists (J.H., J.Q.S). The penicillin was also used for 3 days.

### Follow-up and necropsy

After endoscopic stent placement, all animals were fasted for an additional 24 h. Then, the rabbits were regularly maintained on a semiliquid diet. During follow-up, food-intake and weight were monitored. Because of the tumor growth and the stent insertion, food-intake was less and the rabbits tended to get thinner. Chest CT or endoscopy was neither performed after stent insertion, because the rabbits were too weak to undergo anesthesia or any invasive examination.

A humane endpoint was used in this study, and the rabbits were euthanized 2 weeks after stent implantation. The following lethal injection process was implemented. The animals were intraperitoneally injected with pentobarbital sodium (35 mg/kg) and then injected with 20 ml of air via ear vein.

The esophagus was excised and examined grossly. Photos were obtained to examine the status of the proximal esophageal obstruction. Each stent was gently removed from the esophagus, and endoscopic ultrasound was repeated to examine the tumor size after stent placement in vitro. Then, the esophagus was incised longitudinally. The inner esophagus wall was examined because tumor overgrowth and hyperplasia may repress the stent to the esophageal lumen, thus leading to restenosis. The wall defect was also measured (S = length*width). The size of the esophageal tumor was measured by volume (V = length*width*height/2) and area under EUS (S = length*width). The liver, lung and celiac lymph nodes were examined for tumor metastasis. The tissue samples were stained with hematoxylin-eosin and examined by an experienced GI pathologist. After evaluating the tissue, we fixed the lesion samples in formalin or stored them at -80°C.

In the first part of the study, the stents tended to migrate distally, leading to experiment failure. Thus, the distal edge of the stent was anchored on the esophageal wall surgically.

### Statistical analysis

Data were expressed as means ± SDs. Continuous variables, including food-intake after stent implantation, weight loss and tumor size, were compared using Student’s t-test. Categorical parameters, including gender, proximal esophageal obstruction and metastasis, were compared using a χ^2^ test and Fisher exact test. SPSS version 13.0 (SPSS, Chicago, USA) was used for all statistical analyses. A *P*-value < .05 was considered statistically significant.

## Results

### Stent placement and follow-up

A total of 30 rabbits were prepared in the study for creating tumor model. Severe stricture was observed in 26 rabbits at 2 weeks after tumor implantation, and the remaining 4 rabbits exhibited severe stricture at 3 weeks. At the time of stent inserting, 1 case died in each group because of anesthetic accident, and 1 case in PEMS group died of the esophageal perforation and pneumothorax (*P*>0.05). At the 8^th^ day after stent insertion, 1 rabbit in SEMS group died because of extensive metastasis in both lungs, confirmed by autopsy (*P*>0.05). At the time of sacrifice, migration occurred in 4 cases, 3 in the SEMS group and 1 in the PEMS group (*P*>0.05). All of the rabbits in question were excluded from this study. Thus, a total of 22 rabbits were enrolled into this study (Table 1). For the last 7 animals (3 in the SEMS group and 4 in the PEMS group) in which the stent had been inserted, the abdomen was cut and the distal egde of the stent was anchored to the esophageal wall on the opposite side of the tumor with 5–0 prolene.

**Table 1:** Eight rabbits excluded from the study after stent placement.

	SEMS group	PEMS group	*P*
Migration	3	1	
Death			
Anesthesia	1	1	
procedure	0	1	
Tumor infiltration	1	0	
Total number	5	3	>0.05

### Endoscopic ultrasound findings

In the SEMS group, the average tumor area was 0.33±0.11 cm^2^ before stent placement and 4.73±1.11 cm^2^ after stent placement. In the PEMS group, the average tumor area was 0.30±0.10 cm^2^ before and 1.30±1.06 cm^2^ after. The tumor area measured by EUS was similar between the SEMS and PEMS groups (*P*>0.05) before stent placement but was larger in the SEMS group than in the PEMS group after stent placement (*P*<0.05). In both groups, the tumor area at sacrifice time was larger than that before stent insertion (*P*<0.05).

### Gross and microscopic findings

The lower part of the esophagus was excised from the body. In some specimens, the tumors infiltrated over the serosa membrane, and adhesions were found between the esophagus and the diaphragm or the liver. Gross inspection of the excised specimens revealed no perforation or bleeding in any rabbit.

After endoscopic ultrasound, the esophagus was incised longitudinally. Proximal esophageal obstruction was noted in 1 rabbit in the SEMS group and 1 rabbit in the PEMS group (*P* = 1.00). Minimal or moderate mucosal hyperplasia was observed at both ends of the stented segments in other animals. The granulation and esophageal epithelia at both ends of the stented segments appeared hyperemia.

The average tumor volume was 7.00±4.30 cm^3^ in the SEMS group and 0.94±1.51 cm^3^ in the PEMS group (*P*<0.05). The area of the esophageal wall defect was 0.70±0.63 cm^2^ in the SEMS group and 0.17±0.16 cm^2^ in the PEMS group (*P*<0.05). Gross inspection of the internal surface of the esophagus revealed ulcers, but no bleeding or perforation. The mucosa near the lesion appeared similar in the two groups.

**Fig 3:**
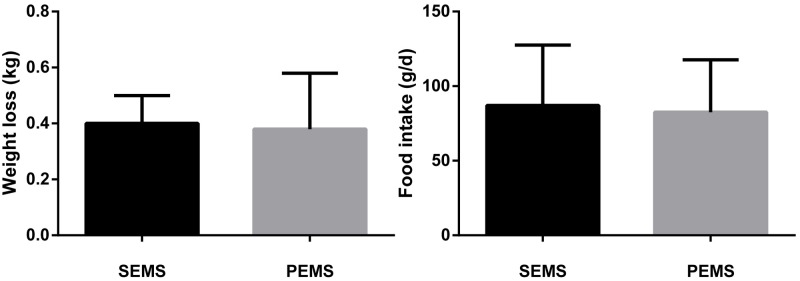
There was no significant difference of weight loss and food intake in the two groups.

**Fig 4:**
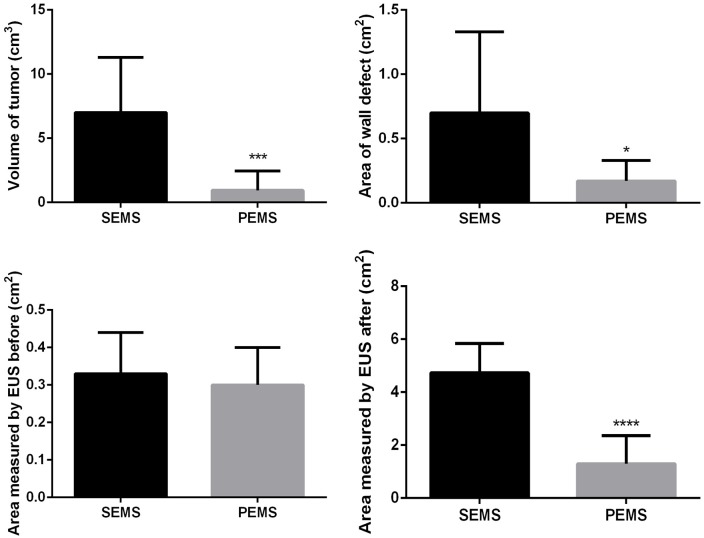
Tumor volume, area of the wall defect, area of the tumor under endoscopic ultrasound before and after stent placement were compared between the SEMS and PEMS group, revealing the tumor size was larger in the SEMS group.

The lungs, liver, kidneys and lymph nodes were inspected. In the SEMS group, lung metastasis was observed in 2 animals, liver metastasis was observed in 3 animals and diaphragm metastasis was observed in 1 animal. In the PEMS group, lung metastasis, lymph node metastasis, and diaphragm metastasis were each observed in 1 animal (*P*>0.05). The characteristics of the animals were described in Figs 3 and 4.

Microscopically, the majority of the lesions were located from the epithelial to the muscular layer. In the proximal and distal parts of the covered stented segments, the average thicknesses of the epithelial layers were similar in both groups. The degree of inflammatory cell infiltration did not differ significantly between the PEMS and SEMS groups.

## Discussion

In most Asian countries, including China, esophageal squamous carcinoma comprises the majority of esophageal cancers. In China, more than 50% patients visiting the doctor have no chance for surgery [17,18]. At present, non-operative palliative treatment by metallic stent insertion is widely used. In recent years, some studies have reported the use of drug-eluting stents in the digestive system, including the esophagus and bile duct, indicating the effect to inhibit tumor progression. For example, Lee et al reported the effect of PEMSs in normal pig bile ducts. The result indicated that PEMSs caused epithelial denudation, mucin hypersecretion and epithelial metaplasia [9]. Furthermore, the researchers proposed that the PEMSs may serve as a new and safe treatment to inhibit tumor growth when inserted for malignant biliary obstruction in humans [19]. However, the next few studies were performed in normal animal models or a small number of human samples, and the results were controversial and could not be generalized [20–22]. Thus, a moderate to large-sized animal model is urgently needed to study the effect of drug-eluting SEMSs [23].

In this study, we chose the New Zealand White rabbits as the model, which were sufficiently large to allow the oral insertion of ultra-slim endoscope, ultrasonic probe and stent introducer set. VX2 carcinoma was an anaplastic squamous cell carcinoma derived from virus-induced papilloma in rabbits proposed by Shope and Hurst in 1993 [24]. This cell can be implanted into any rabbit organ to study tumor formation and progression, which has been widely used to create the cancer of liver, kidney, lung, head and neck[25]. The biological characteristics are similar with that of human carcinoma. Comparing the tumor implantation with VX2 cells, tumor fragments had a higher success rate of tumor growth and lower rate of metastasis [26]. Thus, in our study, we chose the tumor fragments to study the effect of PEMS before the progress of metastasis. The esophageal VX2 tumor model has been rarely been reported by other researchers. In earlier periods of our experiments, the success rate of endoscopic VX2 tumor implantation was as high as 93.7%, thus indicating that our tumor implantation method was safe and reliable. The rabbits developed severe esophageal stricture 2 to 3 weeks after implanting the VX2 fragments, and autopsy examination revealed that the tumor consistently exhibited intra-luminal growth, which imitated advanced human esophageal carcinoma. [16]. Herein, our study was a continuation of the animal study on the success of model building, about the new treatment.

All 22 stents maintained integrity until the time of sacrifice, and only 1 proximal obstruction was observed in each group, which revealed the safety of PEMS and SEMS. Before stent insertion, ultrasound images revealed that tumor size in the two groups was comparable. At the sacrifice time, some significant differences revealed that the tumor size was larger in the SEMS group, including the tumor volume, esophageal wall defect and area under EUS after stent insertion. All of these indices indicated that the PEMS exerted an anti-tumor effect compared with the SEMS. On the other hand, by the time of sacrifice, the tumors had grown in both SEMS and PEMS groups, though tumor growth was slower in the PEMS group. In the PEMS group, the tumor area under EUS was larger after the stent insertion, which revealed that PEMS could only slow the tumor growth but not reduced the tumor volume. These results were consistent with human tumor progression and suggestive of the potential of PEMSs as a therapy. In the future, the drug-eluting stent may reduce the tumor volume by changing different kinds or concentration of drugs.

The mechanism of anti-tumor effect was not clear in this study. In our previous study, PEMS was inserted in normal esophagus in rabbits and the inflammatory cell infiltrating was higher compared with SEMS, but the thickness of esophageal epithelia was not thicker. The PEMS sustaining released drug for at least 6 weeks and stimulated inflammation. The results were consistent with other studies that local drug delivery may inhibit tumor growth by the sustaining histologic changes [27]. However, the molecular mechanism for PEMS needs to be studied in the future.

In our previous experiment, the stent with the diameter of 8mm leaded to discomfort. Given the potential risk of migration, we finally chose the partially covered PEMS with the bare flange at the proximal end, 6 mm in diameter. The relatively smaller diameter of this stent and the different kinds of tissue covered by the stents may partially explain why proximal obstruction was not obvious in the SEMS or PEMS group. However, in the early stage of our experiment, the stents tended to migrate distally (4/19). Thus, after stent insertion for the last 7 animals, we incised the abdomen and anchored the stent to the esophageal wall. These 7 cases lived without incident until their deaths, and no peritoneal metastases were found. The surgical suture of the stent prevented the stent migration and didn’t lead to any complication. The low adverse event rate was acceptable. Tumor adjacent tissue invasion and metastases did not significantly differ in the two groups. At the time of sacrifice, 5 cases in the SEMS group and 3 cases in the PEMS group were evaluated for metastases. However, in our previous study, more metastases were noted when the animals died without stent insertion. After the animal models were created, models started to die at the 4^th^ week after stent insertion [16]. Furthermore, in this study, only one animal died of tumor infiltration at the 8^th^ day after stent insertion. In our opinion, the primary cause was majority animals were sacrificed before metastases formation, for a total tumor-burden time of 4 to 5 weeks (2–3 weeks before stent insertion and 2 weeks after). After stent insertion for 2 weeks, stent removal was not considered. In clinical work, stents are rarely removed in patients with malignant esophageal stricture because of the fast growth of tumor and fibroplasia, let alone the so tiny rabbit esophagus.

This study was to evaluate the effect of paclitaxel eluting SEMS for the esophageal squamous carcinoma. There some wonders of the effect of injection of paclitaxel compared with PEMS. As we knows, the rabbit esophagus is about 4–5 mm in normal cases and even narrower in cancer-bearing animals. Furthermore, the wall of esophagus is thin. After endoscopic injection of the paclitaxel solution, the solution would ooze to the esophageal lumen, adjacent chest or abdomen. The dose of the drug can’t be quantified. The comparing between drug eluting stent and drug injection is inappropriate because of the different drug dose and mechanism. In clinically, injection of drug to treat advanced esophageal carcinoma was rarely reported. There was only one study showed endoscopic injection of mitomycin may release the dysphagia of patients with advanced esophageal carcinoma [28]. No study showed injection of paclitaxel can inhibit the tumor growth. Furthermore, few patients can tolerate repeated endoscopy.

The animal model and stent insertion used in this study cannot be applied to investigate the progression of and therapies for primary esophageal squamous carcinoma. However, our study has its merits. 1. This model imitated the progression of advanced human esophageal cancer. 2. The stent insertion was performed under endoscopy, which is commonly used in humans. 3. We chose to sacrifice the animals 2 weeks after stent implantation and evaluated the anti-tumor effect of PEMSs. In the short term, some tumor progression related death was avoided.

The limitation of this study is that our model could be used only for studying advanced esophageal carcinoma. Additionally, this model has no similarity with human early stage esophageal carcinoma and thus could not be used for studies on endoscopic curative therapies. And, the anti-inflammatory and anti-tumor molecular mechanisms of PEMS need to be confirmed in both cellular and animal levels in future studies.

## Conclusions

In conclusion, this study created a rabbit esophageal squamous carcinoma model under endoscopy and evaluated the effect of PEMSs. SEMS and PEMS are both safe and effective to relieve dysphagia in rabbit esophageal cancer models. And the results revealed that PEMS slowed tumor growth and may be an alternative tool in the management of advanced human esophageal squamous carcinoma, which may provide a new research direction.
